# Impact of Exercise Dose–Response on Maternal Mental Health and Perinatal Depression Prevention: A Systematic Review and Meta–Analysis

**DOI:** 10.3389/ijph.2025.1608940

**Published:** 2025-11-21

**Authors:** Paulina Fuenzalida, Guillermo Droppelmann, Sandra Mahecha, Felipe Feijoo

**Affiliations:** 1 Universidad Mayor Escuela de Medicina, Santiago, Chile; 2 Clínica MEDS, Santiago, Chile; 3 School of Industrial Engineering, Pontificia Universidad Católica de Valparaíso, Valparaíso, Chile

**Keywords:** depression, exercise, perinatal depressive symptoms, physical activity, pregnancy

## Abstract

**Objective:**

To estimate the effect of exercise on perinatal depressive symptoms, focusing on subclinical depression.

**Methods:**

Randomized controlled trials (RCTs) reporting Edinburgh Postnatal Depression Scale (EPDS) scores and evaluating perinatal exercise interventions were eligible. A systematic search was conducted in MEDLINE/PubMed, Web of Science, Scopus, and the Cochrane Library for studies published between 2000 and 2024. Study quality, risk of bias, and heterogeneity were assessed before synthesizing the results using a random-effects model.

**Results:**

Nine RCTs met the inclusion criteria. Exercise significantly reduced depressive symptoms (SMD = −0.47; 95% CI = −0.86 to −0.08; *p* = 0.02) despite high heterogeneity (I^2^ = 88%). Subgroup analyses showed stronger effects during pregnancy (SMD = −0.77; 95% CI = −1.40 to −0.15) than in the postpartum period (SMD = −0.05; 95% CI = −0.31 to 0.22).

**Conclusion:**

Exercise effectively reduces perinatal depressive symptoms and represents a valuable public health intervention. Longer follow-up periods (≥6 months) are needed to confirm the durability of benefits and to evaluate maternal and child outcomes. Future high-quality RCTs with standardized exercise protocols (≥150 min/week of moderate activity) will be essential to translate this evidence into actionable public health and clinical guidelines.

## Introduction

Pregnancy and the postpartum period involve major physiological adaptations across body systems to support fetal development and meet the metabolic demands of gestation [1, 2]. The central nervous system undergoes marked neuroplastic changes, with structural and functional modifications in the maternal brain [3–5]. Functionally, increased vigilance, heightened amygdala activity, and greater emotional sensitivity have been reported [6]. Pregnancy–related reductions in brain volume, particularly a 3% loss in cortical gray matter, affect regions linked to memory and mood regulation [7]. These changes may shift focus from self to caregiving, enhancing responsiveness to newborn needs [6], but also heightening vulnerability to mood disorders due to involvement of emotion–regulating neural networks [3].

Postpartum depression is the most common perinatal mental health disorder, affecting 10%–20% of women [8]. About 8%–12% of pregnant women meet criteria for major depression [9], while among first–time mothers, minor depression affects 11%–20% and major depression 7%–14% [1]. Consequences extend to both mother and child. Maternal stress during pregnancy is linked to altered fetal neurodevelopment, cognitive function, negative affectivity, difficult temperament, and later psychiatric disorders such as sleep problems, attention deficits, and hyperactivity [10].

Nonpharmacological treatments for perinatal mental health disorders have gained attention, with exercise widely studied [11–13]. Maternal exercise promotes neurogenesis and protein expression in offspring, potentially counteracting prenatal stress and enhancing cognitive resilience [10]. In depression, exercise is key, as fatigue increases inactivity risk [14]. Its dose–response relationship in preventing postpartum depression and anxiety is well documented. Psychologically, exercise distracts, reinforces positive behaviors, improves body image, fosters social interaction, and supports emotional well–being [15]. Physiologically, it improves muscle condition, increases beta–endorphin and monoamine production, stimulates Brain–Derived Neurotrophic Factor release, and promotes conversion of kynurenine to kynurenic acid—a neuroprotective metabolite mitigating stress–related brain effects and depression risk [14].

A meta–analysis of 49 prospective studies found an inverse association between physical activity and depression across populations and regions [16]. Despite strong evidence, knowledge gaps remain in perinatal populations. A systematic review on exercise, sedentary behavior, and depressive symptoms in perinatal women found limited preventive evidence, highlighting the need for further research [17]. Another systematic review with meta–analysis in *The Lancet* identified over 20 RCTs on nonpharmacological interventions for depression and anxiety in pregnant women, supporting physical activity as feasible [18]. Another meta–analysis reported a moderate effect of exercise on depression, with greater impact on treatment than prevention [19]. Subgroup analyses comparing pregnant and postpartum women remain inconclusive [20].

Given the impact of depressive symptoms [9], it is essential to examine exercise effects not only in diagnosed perinatal depression but also regarding symptom profiles and exercise characteristics during pregnancy. While exercise reduces depressive symptoms in adults with clinical depression [20], most perinatal research focuses on diagnosed cases, often using cut–off values rather than analyzing outcomes based on Edinburgh Postnatal Depression Scale (EPDS) scores [19]. This leaves a gap in understanding exercise’s role in preventing symptoms in women without prior depression [21, 22].

This study aimed to estimate the effect size of exercise on reducing depressive symptoms during the perinatal period, focusing on subclinical manifestations.

## Methods

### Reporting

This meta–analysis followed the PRISMA 2020 guidelines [23]. The protocol was registered with PROSPERO (CRD420251069284).

### Data Sources

Two coauthors (PF and GD) conducted a systematic search in MEDLINE/PubMed, WOS/Web of Science, SCO–PUS, and the Cochrane Library from 1 January 2000, to 30 September 2024, with disagreements resolved by FF. Full–text access was obtained via institutional subscriptions; gray literature was excluded to focus on peer–reviewed RCTs.

### Search Strategy and Keywords

The search strategy was independently developed by PF and SM (physical medicine specialists) and subsequently validated by GD. It was organized into a matrix using MeSH terms: perinatal period (“Pregnant Women,” “Pregnancy,” “Postpartum Period”), physical activity (“Exercise,” “Physical Activity,” “Exercise Therapy,” “High Intensity Interval Training,” “Motor Activity”), and mental health (“Depression,” “Postpartum Depression,” “Adjustment Disorders,” “Depressive Disorder,” “Dysthymic Disorder”). Boolean operators AND/OR were applied, with filters for human studies, English/Spanish/Portuguese languages, and publications from 1 January 2000, to 30 September 2024.

### Selection Criteria

Women over 18, any race, any level of physical activity, during pregnancy or postpartum (up to 12 months). Participants with diagnosed depression were excluded to focus on subclinical symptoms. Exercise interventions (light to moderate intensity, reported as minutes/week). Groups receiving standard care or other interventions. Depressive symptoms were assessed using EPDS [24].

### Types of Studies

Original, full–length articles reporting randomized controlled trials published in peer–reviewed journals between October 2014 and September 2024 in English, Spanish, or Portuguese were included. Excluded were non–RCT designs (such as observational studies and study protocols), narrative or systematic reviews, letters to the editor, technical reports, and conference abstracts.

### Data Extraction

Two coauthors (PF and GD) manually extracted data under the supervision of SM, with any discrepancies resolved by the senior author. After screening titles and abstracts, full texts of eligible studies were reviewed. Extracted variables, including author, year, country, type and timing of physical activity (pregnancy or postpartum), sample sizes for intervention and control groups, and post–intervention means and SDs, were compiled in an Excel matrix.

### Risk of Bias Assessment

The risk of bias was assessed in all included RCTs using the Cochrane Collaboration risk of bias tool for randomized trials. The evaluation considered bias in the randomization process, deviations from the intended interventions, missing outcome data, outcome measurement, and the selection of reported results. Each factor was rated as low risk, some concerns, or high risk of bias [25]. Visualizations were generated using the Cochrane RoB–2 online tool [26].

### GRADE Assessment

The certainly of the evidence was assessed using GRADEpro GDT software. The GRADE methodology evaluates evidence certainly based on factors such as risk of bias, inconsistency, indirectness, and imprecision, along with additional considerations, categorizing it as low, moderate, or high [27].

### Therapeutic Quality of Exercise Program

The i–CONTENT tool, part of the international consensus on therapeutic exercise and training, was used to assess the therapeutic quality of the exercise program [28]. This instrument enables a clear, systematic evaluation by identifying variations within the intervention. It is based on seven criteria: (1) patient selection, (2) exercise dosage, (3) exercise type, (4) presence of a qualified supervisor, (5) outcome assessment methods and timing, (6) program safety, and (7) adherence to the prescribed regimen. Two reviewers evaluated the program independently, with discrepancies resolved by a third reviewer.

### Statistical Analysis

A random‐effects meta‐analysis was conducted to pool standardized mean differences (SMDs; Cohen’s d) with 95% CIs, thus incorporating both within‐study sampling error and true between‐study heterogeneity [29]. Post‐intervention means and SDs were extracted, and effect sizes were classified using conventional thresholds (0.2 = small; 0.5 = moderate; 0.8 = large).

Subgroup analyses explored differences by pregnancy status (pregnancy vs. postpartum), follow‐up duration (<12 vs. ≥ 12 weeks), average weekly activity time, and intervention timing (during vs. after pregnancy). Forest plots displayed individual and pooled estimates, while funnel plots and Egger’s regression test (p < 0.05) assessed publication bias. Between‐study heterogeneity was quantified with I^2^, with values above 50% indicating substantial heterogeneity.

### Packages and Reports

To perform the meta–analysis of RCTs, the following packages were used in the statistical environment R: meta, metafor, mvmeta and rmeta. A significant level p < 0.05 was established, and 95% confidence intervals (95% CI) were calculated. The results are reported in three decimal places.

All statistical analyzes and graphical representations were performed using R statistical software (version 4.1.3) and Metaanalysisonline.com.

### Ethics Approval

Although ethics committee approval is not explicitly required for systematic reviews and meta–analyses [30], this study only included research that had been approved by an ethics committee and had properly implemented informed consent procedures.

## Results

### Search Results

**FIGURE 1:**
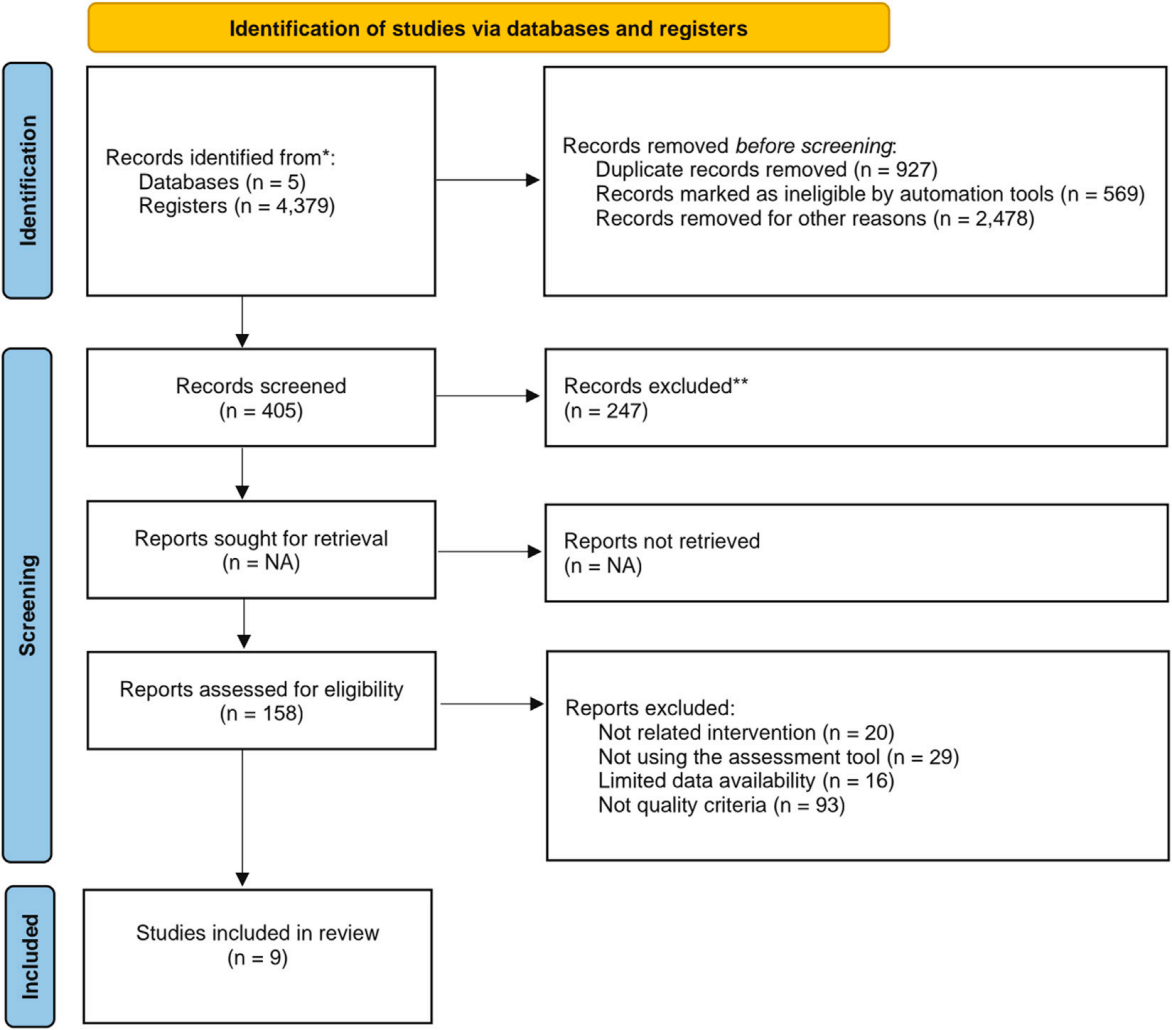
PRISMA 2020 flow diagram (Santiago, Chile. 2025).

Figure 1 presents the flow diagram of the article selection process, following the PRISMA 2020 guidelines [31]. An initial total of 4,379 records was retrieved from database searches after applying the predefined keywords. After removing duplicates and screening titles and abstracts, 405 records were retained for full–text review. Following the application of inclusion and exclusion criteria, 9 randomized controlled trials were included in the final analysis [32–40].

### ROB of Included Studies

**FIGURE 2:**
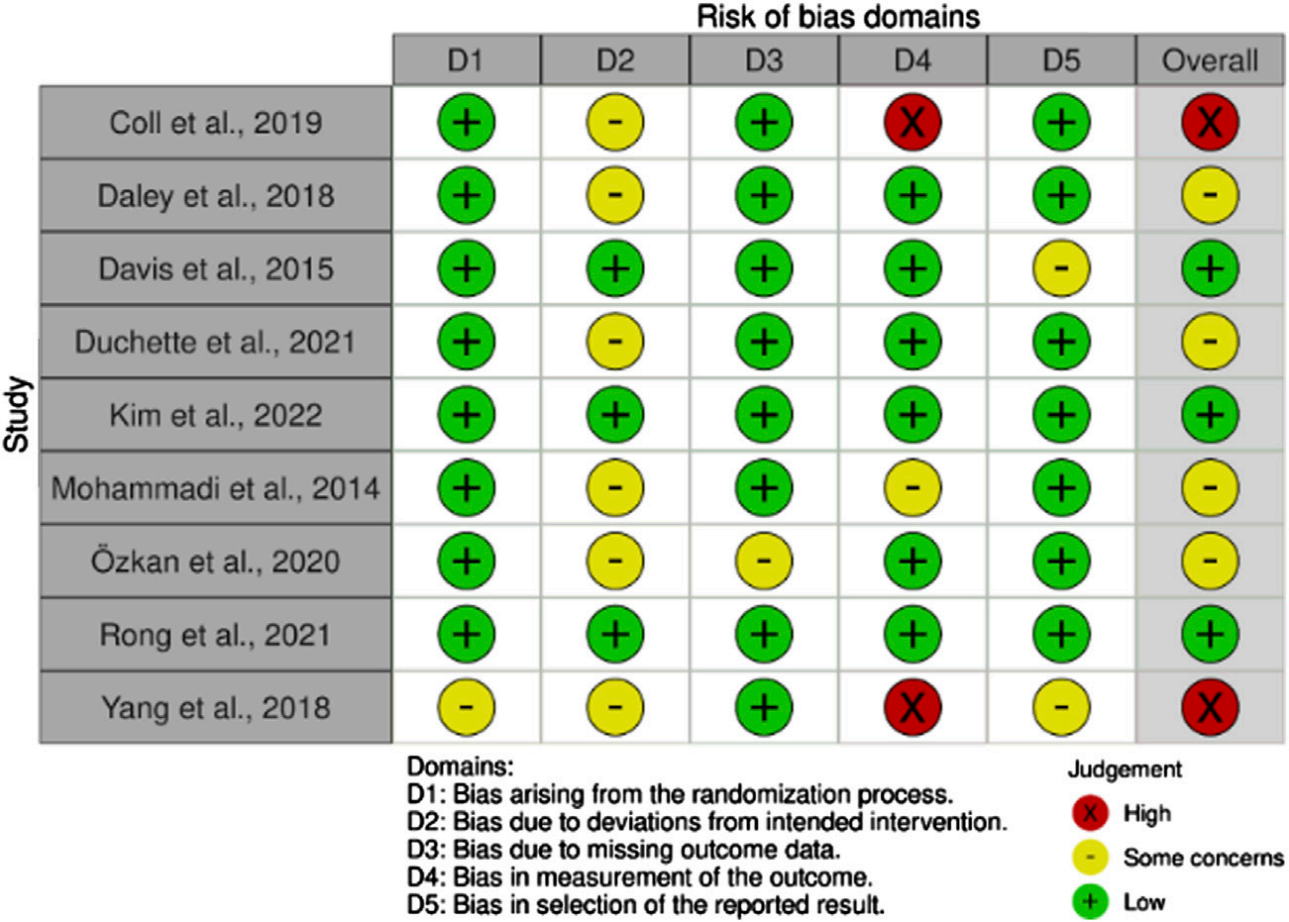
Risk of bias (Santiago, Chile. 2025).

The nine RCTs were assessed using the RoB 2 tool. Overall, three studies were rated as low risk across all domains, four presented some concerns, and two were judged as high risk, primarily due to bias in outcome measurement. Most studies showed a low risk of bias in most domains, although some raised concerns related to outcome measurement and selective reporting. The detailed RoB assessment for each included study is illustrated in Figure 2.

### Study Characteristics and Participants

Research on the impact of physical activity on mental health in pregnant and postpartum women has gained increasing interest in recent years in various regions of the world. The selected studies, published between 2015 and 2021, employed various methodologies to assess the effectiveness of exercise in preventing and managing perinatal depression. In terms of geographical distribution, most studies were conducted in North America, Europe, and Asia, reflecting global interest but a possible geographic imbalance.

### Description of Intervention

The interventions analyzed include low–to moderate–intensity activities such as prenatal yoga, home exercise programs, online Pilates, and aerobic training. Some studies also examined these interventions in pregnant women during the COVID–19 pandemic, a context potentially affecting exercise access, adherence, and psychological vulnerability.

Findings indicate that exercise may help alleviate depression and anxiety symptoms in pregnant women. Improvements in fatigue, sleep quality, and overall, well–being further support incorporating physical activity–based interventions into maternal health strategies. For details on the studies, including objectives, methodologies, and results, see Table 1.

**TABLE 1:** Summary of Exercise Interventions in Perinatal Women. (Santiago, Chile. 2025).

Study	Age (Ex)	Age (Co)	n (Ex)	n (Co)	Frequency (days/week)	Intensity	Time/Duration	Type	Supervision
Coll et al. (Brazil) [32]	27.2 ± 5.5	27.3 ± 5.5	192	387	3	Moderate (RPE)	60 min, 16 weeks	Aerobic + Resistance	S
Daley et al. (UK) [33]	27.5 ± 6.3	27.5 ± 6.3	189	194	2	Moderate (NR)	30 min, 8 weeks	Aerobic	S
Davis et al. (USA) [34]	29.74 ± 5.40	30.57 ± 4.46	20	19	1	NR	75 min, 8 weeks	Yoga (Ashtanga Vinyasa)	S
Duchette et al. (USA) [35]	28.52 ± 3.74	28.52 ± 3.74	10	9	1	NR	75 min, 10 weeks	Prenatal Yoga	S
Kim et al. (South Korea) [36]	NR	NR	8	8	2	Moderate (%HRmax and RPE)	50 min, 8 weeks	Pilates	S
Mohammadi et al. (Iran) [37]	25	25	36	36	3	Light (NR)	20–30 min, 12–18 weeks	Flexibility + Breathing	US
Özkan et al. (Turkey) [38]	26.7 ± 5.34	27.1 ± 5.28	34	31	5	Light to Vigorous (NR)	30 min, 4 weeks	Aerobic + Resistance + Flexibility	S
Rong et al. (China) [39]	29.00 ± 2.81	28.16 ± 2.78	32	32	3	Moderate (NR)	60 min, 12 weeks	Yoga	S
Yang et al. (Taiwan) [40]	31.89	32.45	64	65	3	Moderate (NR)	15 min, 12 weeks	Aerobic	US

NR, Not reported; RPE, Rating of Perceived Exertion; HRmax, Maximum Heart Rate; S, Supervised; US, Unsupervised.

### Frequency

Two studies [34, 35] reported conducting exercise training once a week. Two others [33, 36] implemented sessions twice per week. Four studies [32, 37, 39, 40] documented a frequency of three times per week, while only one study [38] applied the intervention five times per week.

### Intensity

In two of the nine studies [34, 35], the intensity of exercise was not specified. Only one article reported light–intensity training [37], while the remaining studies applied moderate–intensity protocols [32, 33, 38–40], with one implementing a progressive intensity approach from light to vigorous levels [38]. Intensity was based on ACOG recommendations in five studies [32, 33, 36, 38, 40], and on the 2019 Canadian Guideline for Physical Activity Throughout Pregnancy in one study [39]. Although seven studies specified intensity, and all moderate–intensity trials justified their choice, only three [32, 33, 36] described how intensity was measured (via perceived–exertion scales or percentage of maximum heart rate).

### Time and Duration

The included studies reported session durations ranging from 15 to 75 min per session, with total intervention periods spanning 4 to 18 weeks.

### Type

Four studies implemented an aerobic exercise program [32, 33, 38, 40], two of which combined aerobic and resistance training [32, 38]. Three studies described the intervention as yoga–based [34, 35, 39] and one as Pilates [36]. Furthermore, two studies incorporated flexibility–focused exercises [37, 38]. Regarding pregnancy–specific adaptations, four studies [34–36, 39] reported implementing modifications or addressing safety considerations.

### Progression

Five studies reported exercise progression [32, 33, 36, 38, 40]. In most cases [32, 33, 38], progression was guided by the supervisor’s subjective evaluation of each participant’s physical condition [36]. One study noted progression without specifying the method [40].

### Supervision

Only two studies [37, 40] did not include supervision of the sessions, and one study [38] supervised only the initial session. Among the supervised interventions, one was conducted synchronously through online video calls [36]. The format of supervision, individual vs. group–based, is relevant as it may influence the adherence and participation of the participants. Five studies implemented group–based interventions [32, 34–36, 39].

### Therapeutic Quality of the Exercise Programs

In the quality assessment, three items did not achieve the highest rating: qualified supervision, program safety, and adherence to the intervention. Regarding safety, five of the nine studies provided insufficient information. Of the remaining four, two explicitly reported no serious or unexpected adverse events during the intervention [32, 39]. In Davis et al., a preterm birth at 24.5 weeks was noted but not linked to exercise. Daley et al. was the only study to report adverse events, though details were missing, and their frequency was similar in both groups [33].

Adherence was the main methodological limitation, potentially affecting the reliability of findings. Only two trials reached the ≥70% attendance threshold [34, 39]. Coll et al. reported 40.4% meeting this criterion [32], Mohammadi et al. found 67% attended fewer than half the sessions [37], and Daley et al. reported a median attendance of 28.5%. Three studies [35, 36, 38] tracked attendance via supervision or self–report but did not provide specific data. Detailed results are shown in Supplementary File 1.

### Average Effect

**FIGURE 3:**
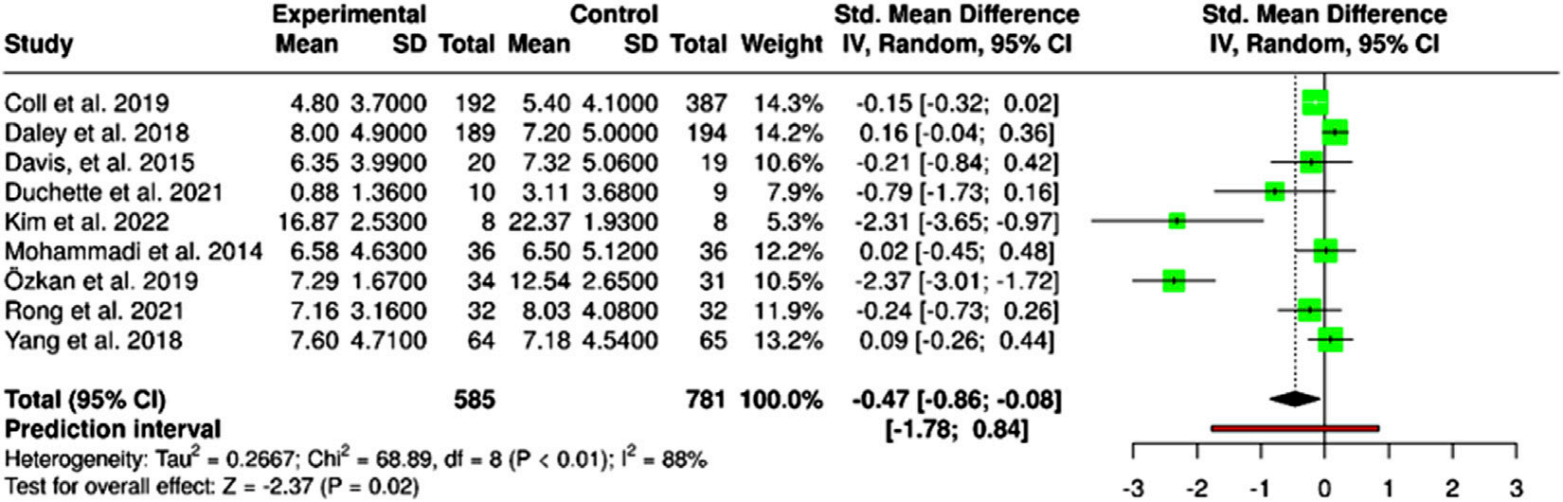
Forest plot of standardized mean differences (Santiago, Chile. 2025).

The meta–analysis results, shown in Figure 3, present the standardized mean differences (SMD) between experimental and control groups in the included studies. Effect sizes ranged from −2.37 to 0.16, indicating substantial heterogeneity. The high heterogeneity (I^2^ = 88%, χ^2^ = 68.89, P < 0.01) suggests variability between studies is unlikely due to chance and may reflect differences in methodology, sample characteristics, or intervention context.

The prediction interval (−1.78 to 0.84) illustrates the range of effects expected in future studies, from a clinically significant benefit to no observable effect. The pooled effect size indicates a moderate but statistically significant reduction in depressive symptoms (SMD = −0.47; 95% CI: −0.86 to −0.08; Z = −2.37; P = 0.02). However, given the heterogeneity, these findings should be interpreted cautiously, considering possible clinical and methodological moderators influencing both magnitude and direction of the effect.

Supplementary File 2 shows a funnel plot assessing potential publication bias in the included studies. The asymmetric distribution of points suggests a possible lack of studies with negative or nonsignificant effects, a common sign of publication bias. While most studies cluster on the right side of the no–effect line (SMD = 0), a few extreme values on the left may indicate substantial heterogeneity. The wider dispersion of studies with higher standard errors suggests that effects in smaller studies may be less precise or influenced by methodological variability. These observations highlight the need for cautious interpretation, as effect sizes may be overestimated due to missing unpublished studies with null or opposing results.

### Subgroups Analysis Based on Intervention Type

**FIGURE 4:**
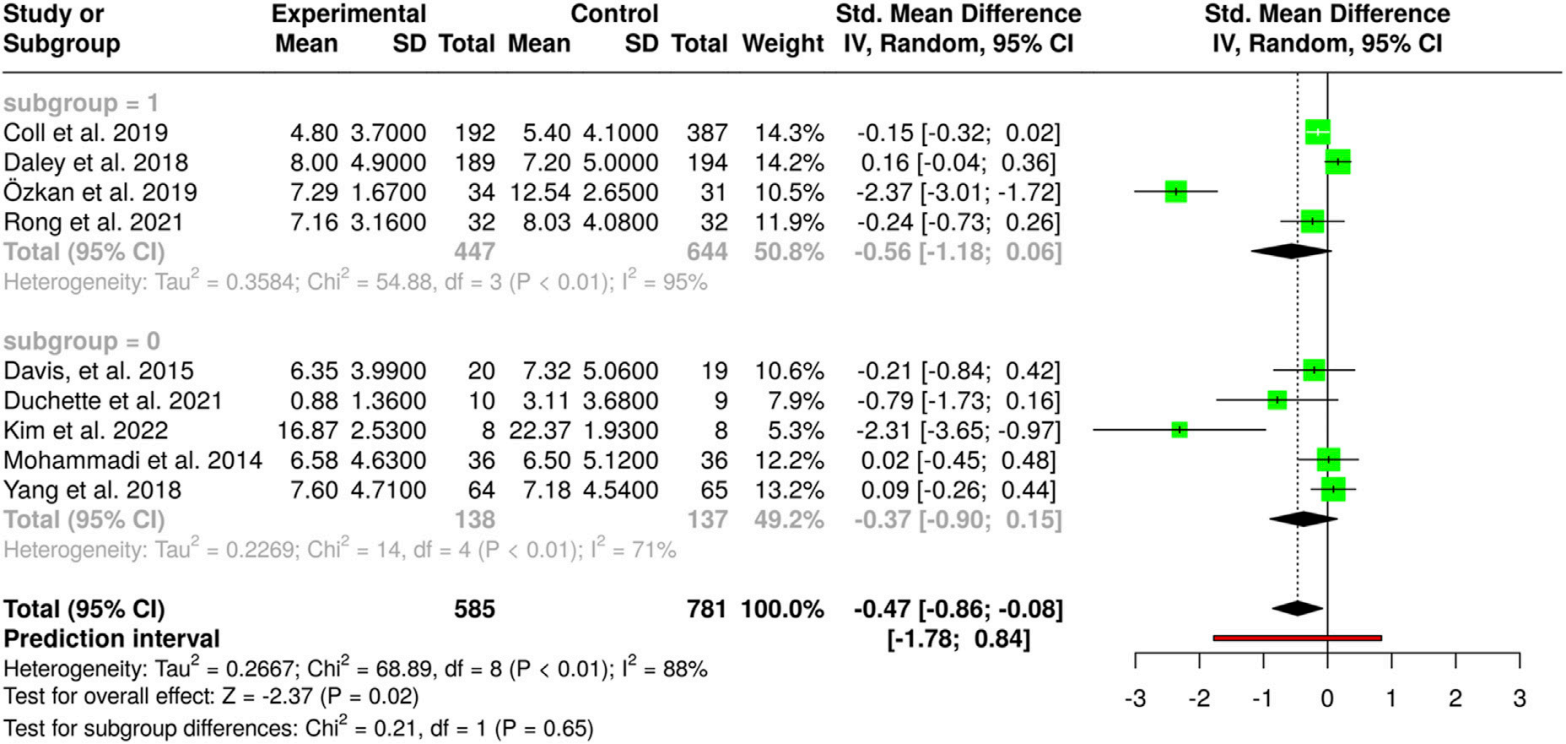
Subgroup analysis based on intervention type, comparing studies conducted during pregnancy versus postpartum (Santiago, Chile. 2025).

Figure 4 presents a subgroup (0 and 1) analysis categorizing studies by relevant methodological or clinical characteristics. Heterogeneity differs notably between subgroups, with I^2^ = 94% in the first and only 9% in the second, suggesting variability is concentrated in a specific subset. The overall effect in the first subgroup is larger and statistically significant (SMD = −0.77; 95% CI –1.40 to −0.15), whereas in the second it is smaller and not significant (SMD = −0.05; 95% CI –0.31 to 0.22). The test for subgroup differences (χ^2^ = 4.44, P = 0.04) indicates that this stratification explains part of the total heterogeneity. These findings underscore the importance of considering methodological and clinical factors when interpreting results, as effect magnitude and consistency can vary across populations or study designs.

### Subgroups Analysis Based on Sources of Heterogeneity

**FIGURE 5:**
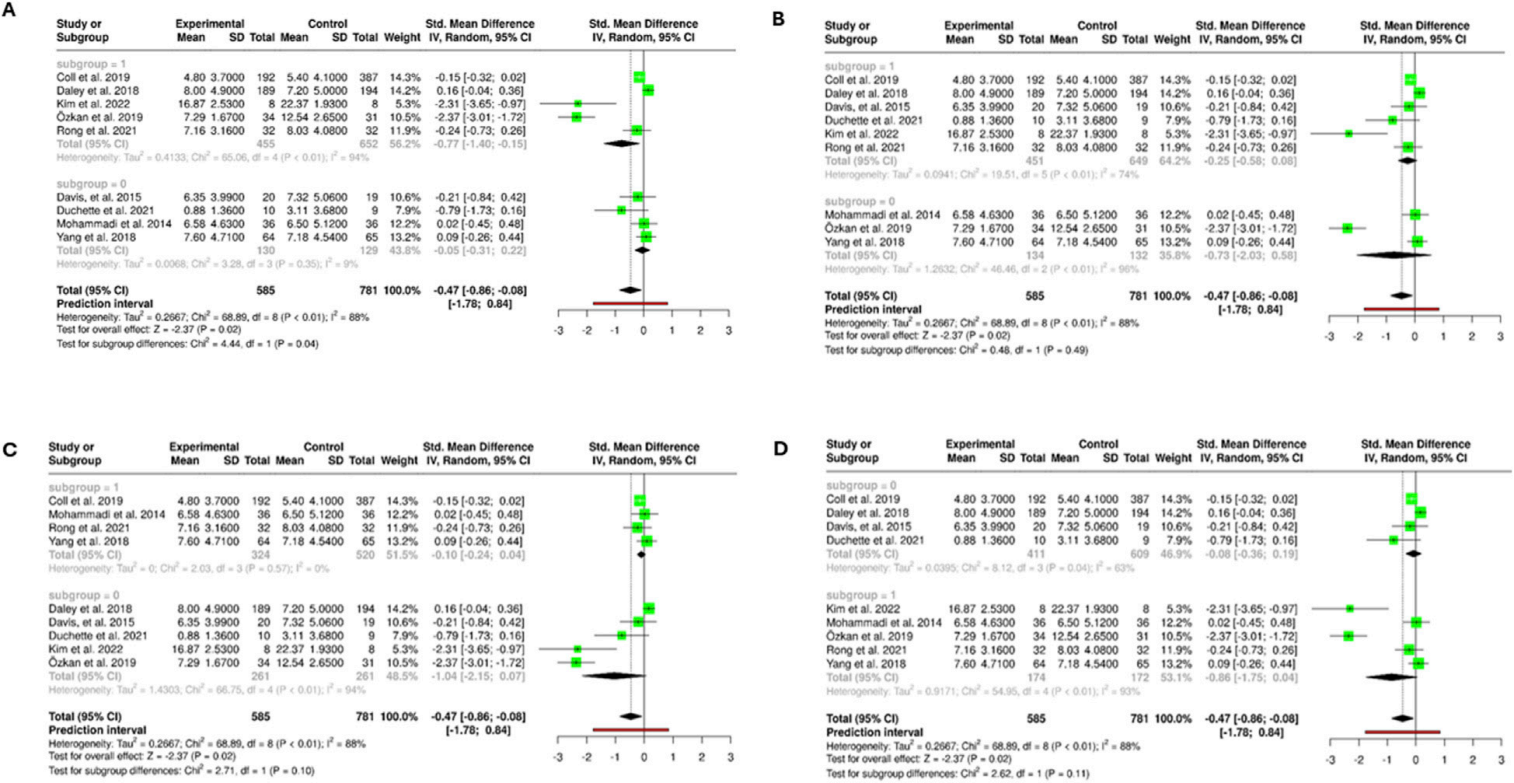
Subgroup meta-analyses exploring sources of heterogeneity. **(A)** Analyzes outcomes by follow-up duration (<12 vs. ≥12 weeks). **(B)** Stratifies results according to exercise supervision (supervised vs. unsupervised programs). **(C)** Categorizes interventions by intensity (light/moderate vs. vigorous). **(D)** Compares studies based on weekly activity dose (<150 vs. ≥150 min) (Santiago, Chile. 2025).

Figure 5A heterogeneity differs between subgroups, with I^2^ = 95% in the first and 71% in the second, indicating greater variability in the first subgroup. The first subgroup shows a moderate negative trend (SMD = −0.56; 95% CI –1.18 to 0.06), not statistically significant. The second subgroup has a smaller, also nonsignificant effect (SMD = −0.37; 95% CI –0.90 to 0.15). The test for subgroup differences (χ^2^ = 0.21, P = 0.65) suggests the stratification variable does not explain a relevant portion of heterogeneity. Overall, the meta–analytic effect is statistically significant (SMD = −0.47; 95% CI –0.86 to −0.08; P = 0.02), though the wide prediction interval (−1.78 to 0.84) reflects uncertainty in future estimates. These results stress the importance of considering clinical and methodological variability when interpreting pooled effects.

Figure 5B this subgroup analysis assesses variability by methodological or clinical characteristics. The first subgroup shows moderate heterogeneity (I^2^ = 74%), while the second has higher heterogeneity (I^2^ = 96%), suggesting important differences in result consistency. The first subgroup effect (SMD = −0.25; 95% CI –0.58 to 0.08) is not significant, whereas the second is more pronounced but uncertain (SMD = −0.73; 95% CI –2.03 to 0.58). The test for subgroup differences (χ^2^ = 0.48, P = 0.49) shows no significant difference, indicating this stratification does not explain a relevant part of total heterogeneity.

Figure 5C this analysis explores potential heterogeneity sources. The first subgroup shows no detectable heterogeneity (I^2^ = 0%, χ^2^ = 2.03, P = 0.57), indicating a consistent effect (SMD = −0.10; 95% CI –0.24 to 0.04). The second subgroup presents substantial heterogeneity (I^2^ = 94%, χ^2^ = 66.75, P < 0.01), with significant variability (SMD = −1.04; 95% CI –2.15 to 0.07). The test for subgroup differences (χ^2^ = 2.71, P = 0.10) shows no significant difference, suggesting stratification may not fully account for the heterogeneity.

Figure 5D this analysis further explores heterogeneity sources. The first subgroup has moderate heterogeneity (I^2^ = 63%, χ^2^ = 8.12, P = 0.04) and a nonsignificant effect (SMD = −0.08; 95% CI –0.36 to 0.19). The second subgroup shows substantial heterogeneity (I^2^ = 93%, χ^2^ = 54.95, P < 0.01) with a more pronounced, though nonsignificant, effect (SMD = −0.86; 95% CI –1.75 to 0.04). The test for subgroup differences (χ^2^ = 2.62, P = 0.11) indicates no significant difference, suggesting stratification may not fully explain overall heterogeneity.

### GRADE Assessment

The analysis suggests that, while most studies indicate a beneficial effect of exercise compared to control, the strength of the evidence varies. Studies with high certainty (e.g., Daley et al., Davis et al., Özkan et al.) provide more reliable results, while those with low or very low certainty (e.g., Coll et al.) should be interpreted with caution. The impact of exercise appears to be stronger in studies with robust associations and larger effect sizes. The summary of results in Supplementary Files 3, 4.

## Discussion

This meta–analysis examines the preventive effect of exercise on subclinical perinatal depressive symptoms and underscores its importance as a scalable public health strategy to improve maternal mental health.

### Effect of Exercise on Perinatal Depressive Symptoms

Pooling standardized mean differences (Cohen’s d) revealed a significant overall reduction in depressive symptoms during the perinatal period. Subgroup analyses, by pregnancy versus postpartum, follow–up (<12 vs. ≥ 12 weeks), weekly activity volume, and timing, highlighted key moderators despite substantial heterogeneity. These findings support personalized exercise prescriptions.

Our results align with guidelines endorsing physical activity in pregnancy and postpartum for maternal and fetal benefits: improved endocrine and glycemic control, lower triglycerides, prevention of excessive gestational weight gain, and facilitated postpartum weight loss [41–51]. Regular exercise also shortens labour, reduces obstetric complications, and enhances perinatal outcomes [52], while alleviating stress, anxiety, and insomnia [46, 53].

The Developmental Origins of Health and Disease (DOHaD) hypothesis [54], extended to mental health, suggests prenatal stress can impair fetal neurodevelopment and predispose to later psychiatric disorders [55]. Infants of mothers with antenatal depressive symptoms exhibit poorer behavioural regulation and autonomic stability [56], so maternal exercise may protect both mother and child.

### Public Health Implications and Implementation

These data advocate integrating structured exercise into perinatal care, with clear frequency and intensity guidelines in national protocols and provider training [57, 58]. To ensure equity, programs should address geographic, socioeconomic, and cultural barriers via community partnerships, telehealth, and subsidized classes [59, 60]. Although formal cost–benefit analyses are pending, preliminary evidence suggests preventive exercise could lower downstream perinatal depression costs. Real–world monitoring through registries and EPDS screening will inform policy refinement [61, 62].

### Methodological Considerations and Reporting Standards

Variable subgroup outcomes underscore the need for standardized intervention reporting. Tools such as i–CONTENT [28] and CERT (FITT–Pro framework) [63, 64] enhance reproducibility and clinical relevance, though only CERT details exercise delivery parameters. Intervention quality appraisal should guide tool selection.

Few studies detailed pregnancy‐specific adaptations: Davis et al. noted modifications without specifics; Duchette et al. avoided certain positions [35]; Rong et al. adapted yoga for pregnancy [39]; Kim et al. incorporated rest intervals [36, 65, 66].

Low adherence, driven by perceived fetal risk and postpartum time constraints [67–69], highlights the need for maternal education, reassurance about exercise safety, and support services (e.g., childcare, accessible programs).

Debate has shifted from “whether” to “how” perinatal exercise should be prescribed [70]. While guidelines recommend 150 min/week [71, 72], our analysis suggests benefits at 100 min, indicating that optimal intensity and volume warrant further study and individualized recommendations, as urged by Dalbo et al. [64].

A key limitation in the broader literature is the heterogeneity of depression assessment tools (EPDS, CES-D, HADS, BDI, HAMA) [19, 73, 74], which complicates comparability across studies. However, in our meta-analysis, only trials using the EPDS were included to ensure methodological consistency. Although EPDS remains the most widely applied scale, it may underestimate exercise effects [56, 75].

Despite the consistent direction of benefit, the high heterogeneity (I^2^ = 88%) observed across trials reflects substantial methodological and clinical variability. This dispersion likely stems from differences in exercise modalities (aerobic, yoga, Pilates), supervision formats (in-person vs. online), intervention durations (4–18 weeks), and participant characteristics such as baseline EPDS scores and gestational stage. Subgroup analyses by timing (pregnancy vs. postpartum) and intervention partially explained this variability, indicating that exercise during pregnancy and longer, supervised programs produced more consistent effects. However, residual heterogeneity suggests that contextual and behavioral factors, including cultural attitudes toward exercise, adherence rates, and perceived safety, may influence outcomes. These findings highlight the need for future RCTs to adopt standardized protocols and detailed reporting frameworks (CERT, i-CONTENT) to improve comparability and refine dose–response estimations.

### Limitations

A limited number of studies with high methodological heterogeneity (I^2^ > 50%) and some at high or unclear risk of bias may affect reliability. Potential publication bias was suggested by funnel–plot asymmetry. Variability in intervention protocols constrained deeper subgroup and sensitivity analyses. Finally, maternal mental health encompasses subclinical symptoms—anxiety, stress, fatigue, insomnia—that often precede formal depression [76–79]. By addressing depressive symptoms broadly rather than diagnoses alone, this meta–analysis captures real–world emotional well–being and fills a critical methodological gap.

### Conclusion

These results support the integration of physical activity into perinatal mental health strategies, as our meta–analysis found a significant reduction in depressive symptoms among women who exercised during the perinatal period. However, the high heterogeneity and methodological limitations of the included studies warrant a cautious interpretation of these findings. Furthermore, promoting physical activity during pregnancy may plausibly have positive effects on fetal neurodevelopment, potentially reducing the risk of emotional and behavioral disorders in later life, although this hypothesis should be explored in future research. Finally, improving the quality of exercise–based interventions through standardized tools such as i–CONTENT or CERT is essential to strengthen the evidence base and optimize clinical implementation.

## References

[1] Barba–Müller E Craddock S Carmona S Hoekzema E . Brain Plasticity in Pregnancy and the Postpartum Period: Links to Maternal Caregiving and Mental Health. Arch Women’s Ment Health (2019) 22:289–99. 10.1007/s00737-018-0889-z 30008085 PMC6440938

[2] Donofry SD Jouppi RJ Call CC Kolko Conlon RP Levine MD . Improvements in Maternal Cardiovascular Health Over the Perinatal Period Longitudinally Predict Lower Postpartum Psychological Distress Among Individuals Who Began Their Pregnancies with Overweight or Obesity. J Am Heart Assoc (2024) 13:e034153. 10.1161/JAHA.123.034153 38874183 PMC11255758

[3] Maguire J McCormack C Mitchell A Monk C . Neurobiology of Maternal Mental Illness. Handbook Clin Neurol (2020) 171:97–116. 10.1016/B978-0-444-64239-4.00005-9 32736761

[4] Servin–Barthet C Martínez–García M Pretus C Paternina–Die M Soler A Khymenets O The Transition to Motherhood: Linking Hormones, Brain and Behaviour. Nat Rev Neurosci (2023) 24:605–19. 10.1038/s41583-023-00733-6 37612425

[5] Carmona S Martínez–García M Paternina–Die M Barba–Müller E Wierenga LM Alemán–Gómez Y Pregnancy and Adolescence Entail Similar Neuroanatomical Adaptations: A Comparative Analysis of Cerebral Morphometric Changes. Hum Brain Mapp (2019) 40:2143–52. 10.1002/hbm.24513 30663172 PMC6865685

[6] Van den Bergh BR Heuvel MI Lahti M Braeken M de Rooij SR Entringer S Prenatal Developmental Origins of Behavior and Mental Health: The Influence of Maternal Stress in Pregnancy. Neurosci and Biobehavioral Rev (2020) 117:26–64. 10.1016/j.neubiorev.2017.07.003 28757456

[7] Pownall M Hutter RR Rockliffe L Conner M . Memory and Mood Changes in Pregnancy: A Qualitative Content Analysis of Women’s First–Hand Accounts. J Reprod Infant Psychol (2023) 41:516–27. 10.1080/02646838.2022.2052827 35306947

[8] Banti S Mauri M Oppo A Borri C Rambelli C Ramacciotti D From the Third Month of Pregnancy to 1 Year Postpartum. Prevalence, Incidence, Recurrence, and New Onset of Depression. Results from the Perinatal Depression–Research and Screening Unit Study. Compr Psychiatry (2011) 52:343–51. 10.1016/j.comppsych.2010.08.003 21683171

[9] Hutner LA Catapano LA Nagle–Yang SM Williams KE Osborne LM . Textbook of Women’s Reproductive Mental Health. American Psychiatric Pub (2021).

[10] Kim TW Park SS Kim SH Kim MK Shin MS Kim SH . Exercise Before Pregnancy Exerts Protective Effect on Prenatal Stress–Induced Impairment of Memory, Neurogenesis, and Mitochondrial Function in Offspring. J Exerc Rehabil (2024) 20:2–10. 10.12965/jer.2448068.034 38433854 PMC10902695

[11] Gallo–Galán L Gallo–Vallejo M Gallo–Vallejo J . Evidence Based Medicine (EBM). *J Phys Exercise pregnancy* *SEMERGEN* (2022) 48:423–30. 35527186 10.1016/j.semerg.2022.02.008

[12] McGrath A Lambe B Matthews E McDonnell K Harrison M Kehoe B . Determinants of Physical Activity Promotion in Primary Care from the Patient Perspective of People at risk of or Living with Chronic Disease: A COM-B analysis. BMC Prim Care (2024) 25:190 38807071 10.1186/s12875-024-02440-2 PMC11134685

[13] Yu H Santos–Rocha R Radzimin´ski Ł Jastrze˛bski Z Bonisławska I Szwarc A Effects of 8–week Online, Supervised High–Intensity Interval Training on the Parameters Related to the Anaerobic Threshold, Body Weight, and Body Composition During Pregnancy: A Randomized Controlled Trial. Nutrients (2022) 14:5279. 10.3390/nu14245279 36558438 PMC9781372

[14] Mahecha S . Actividad Física Y Ejercicio En Salud Y Enfermedad. In: Santiago, Chile: Editorial Mediterráneo (2017).

[15] Harrison CL Thompson RG Teede HJ Lombard CB . Measuring Physical Activity During Pregnancy. Int J Behav Nutr Phys Activity (2011) 8:19–8. 10.1186/1479-5868-8-19 21418609 PMC3069935

[16] Schuch FB Vancampfort D Firth J Rosenbaum S Ward PB Silva ES Physical Activity and Incident Depression: A Meta–Analysis of Prospective Cohort Studies. Am J Psychiatry (2018) 175:631–48. 10.1176/appi.ajp.2018.17111194 29690792

[17] Teychenne M York R . Physical Activity, Sedentary Behavior, and Postnatal Depressive Symptoms: A Review. Am J Prev Med (2013) 45:217–27. 10.1016/j.amepre.2013.04.004 23867030

[18] Zeng G Niu J Zhu K Li F Li L Gao K Effects of Non–Pharmacological Interventions on Depressive and Anxiety Symptoms in Pregnant Women: A Systematic Review and Network Meta–Analysis. EClinicalMedicine (2025) 79:103011. 10.1016/j.eclinm.2024.103011 39802308 PMC11718295

[19] Ji M Li R Xu Y . Meta–Analysis of the Effect of Different Exercise Modalities in the Prevention and Treatment of Perinatal Depression. J affective Disord (2024) 350:442–51. 10.1016/j.jad.2024.01.076 38228277

[20] He L Soh KL Huang F Khaza’ai H Geok SK Vorasiha P The Impact of Physical Activity Intervention on Perinatal Depression: A Systematic Review and Meta–Analysis. J affective Disord (2023) 321:304–19. 10.1016/j.jad.2022.10.026 36374719

[21] Morres ID Tzouma NA Hatzigeorgiadis A Krommidas C Kotronis KV Dafopoulos K Exercise for Perinatal Depressive Symptoms: A Systematic Review and Meta–Analysis of Randomized Controlled Trials in Perinatal Health Services. J Affective Disord (2022) 298:26–42. 10.1016/j.jad.2021.10.124 34728280

[22] Yan LB Zhang JZ Zhou Q Peng FL . Multidimensional Analyses of the Effect of Exercise on Women with Depression: A Meta–Analysis. Medicine (2021) 100:e26858. 10.1097/MD.0000000000026858 34414936 PMC8382388

[23] Page MJ McKenzie JE Bossuyt PM Boutron I Hoffmann TC Mulrow CD The PRISMA 2020 Statement: An Updated Guideline for Reporting Systematic Reviews. bmj (2021) 372:n71. 10.1136/bmj.n71 33782057 PMC8005924

[24] Cox JL Holden JM Sagovsky R . Detection of Postnatal Depression: Development of the 10–Item Edinburgh Postnatal Depression Scale. The Br J Psychiatry (1987) 150:782–6. 10.1192/bjp.150.6.782 3651732

[25] Higgins JP Savovic´ J Page MJ Elbers RG Sterne JA . Assessing Risk of Bias in a Randomized Trial. Cochrane handbook Syst Rev Interventions (2019) 205–28. 10.1002/9781119536604.ch8

[26] Sterne JA Savovic´ J Page MJ Elbers RG Blencowe NS Boutron I RoB 2: A Revised Tool for Assessing Risk of Bias in Randomised Trials. BMJ (2019) 366. 10.1136/bmj.l4898 31462531

[27] Evidence Prime I . Gradepro GDT: Gradepro Guideline Development Tool. Hamilton, ON: McMaster University (2015). p. 140. [Software].

[28] Hoogeboom TJ Kousemaker MC Van Meeteren NL Howe T Bo K Tugwell P i–CONTENT Tool for Assessing Therapeutic Quality of Exercise Programs Employed in Randomised Clinical Trials. Br J Sports Med (2021) 55:1153–60. 10.1136/bjsports-2019-101630 33144350 PMC8479742

[29] Kanters S . Fixed–And Random–Effects Models. Meta–Research: Methods Protoc (2022) 2345:41–65. 10.1007/978-1-0716-1566-9_3 34550583

[30] Harriss DJ MacSween A Atkinson G . Ethical Standards in Sport and Exercise Science Research: 2020 Update. Int J Sports Med (2019) 40:813–17. 31614381 10.1055/a-1015-3123

[31] Haddaway NR Page MJ Pritchard CC McGuinness LA . PRISMA2020: An R Package and Shiny App for Producing PRISMA 2020– Compliant Flow Diagrams, with Interactivity for Optimised Digital Transparency and Open Synthesis. Campbell Syst Rev (2022) 18:e1230. 10.1002/cl2.1230 36911350 PMC8958186

[32] Coll CVN Domingues MR Stein A da Silva BGC Bassani DG Hartwig FP Efficacy of Regular Exercise During Pregnancy on the Prevention of Postpartum Depression: The PAMELA Randomized Clinical Trial. JAMA Netw open (2019) 2:e186861. 10.1001/jamanetworkopen.2018.6861 30646198 PMC6324311

[33] Daley A Riaz M Lewis S Aveyard P Coleman T Manyonda I Physical Activity for Antenatal and Postnatal Depression in Women Attempting to Quit Smoking: Randomised Controlled Trial. BMC Pregnancy and Childbirth (2018) 18:156–10. 10.1186/s12884-018-1784-3 29747597 PMC5946409

[34] Davis K Goodman SH Leiferman J Taylor M Dimidjian S . A Randomized Controlled Trial of Yoga for Pregnant Women with Symptoms of Depression and Anxiety. Complement Therapies Clin Pract (2015) 21:166–72. 10.1016/j.ctcp.2015.06.005 26256135

[35] Duchette C Tolusso DV Stone WJ Blankenship MM Tinius RA . Prenatal Yoga and Mental Health During the COVID–19 Pandemic: A Randomized–Control Trial. OBM Integr Complimentary Med (2021) 6:1–17. 10.21926/obm.icm.2104051 35287283 PMC8918019

[36] Kim HB Hyun AH . Psychological and Biochemical Effects of an Online Pilates Intervention in Pregnant Women During COVID–19: A Randomized Pilot Study. Int J Environ Res Public Health (2022) 19:10931. 10.3390/ijerph191710931 36078648 PMC9517892

[37] Mohammadi F Malakooti J Babapoor J Mohammad–Alizadeh–Charandabi S . The Effect of a Home–Based Exercise Intervention on Postnatal Depression and Fatigue: A Randomized Controlled Trial. Int J Nurs Pract (2015) 21:478–85. 10.1111/ijn.12259 24620734

[38] Özkan SA Kücükkelepce DS Korkmaz B Yılmaz G Bozkurt MA . The Effectiveness of an Exercise Intervention in Reducing the Severity of Postpartum Depression: A Randomized Controlled Trial. Perspect Psychiatr Care (2020) 56:844–50. 10.1111/ppc.12500 32187390

[39] Rong L Wang R Ouyang YQ Redding SR . Efficacy of Yoga on Physiological and Psychological Discomforts and Delivery Outcomes in Chinese Primiparas. Complement Therapies Clin Pract (2021) 44:101434. 10.1016/j.ctcp.2021.101434 34175716

[40] Yang CL Chen CH . Effectiveness of Aerobic Gymnastic Exercise on Stress, Fatigue, and Sleep Quality During Postpartum: A Pilot Random– Ized Controlled Trial. Int J Nurs Stud (2018) 77:1–7. 10.1016/j.ijnurstu.2017.09.009 28950158

[41] Hayman M Brown WJ Brinson A Budzynski–Seymour E Bruce T Evenson KR . Public Health Guidelines for Physical Activity During Pregnancy from Around the World: A Scoping Review. Br J Sports Med (2023) 57:940–7. 10.1136/bjsports-2022-105777 36604155

[42] Evenson KR Barakat R Brown WJ Dargent–Molina P Haruna M Mikkelsen EM Guidelines for Physical Activity During Pregnancy: Comparisons from Around the World. Am J lifestyle Med (2014) 8:102–21. 10.1177/1559827613498204 25346651 PMC4206837

[43] Evenson KR Brown WJ Brinson AK Budzynski–Seymour E Hayman M . A Review of Public Health Guidelines for Postpartum Physical Activity and Sedentary Behavior from Around the World. J Sport Health Sci (2024) 13:472–83. 10.1016/j.jshs.2023.12.004 38158180 PMC11184298

[44] Kwiatkowska E Kajdy A Sikora–Szubert A Karowicz–Bilinska A Zembron–Lacny A Ciechanowski K Polish Society of Gynecologists and Obstetricians (PTGiP) and Polish Society of Sports Medicine (PTMS) Recommendations on Physical Activity During Pregnancy and the Postpartum Period. Ginekologia Polska (2024) 95:218–31. 10.5603/GP.a2023.0080 37599577

[45] Gervais MJ Ruchat SM Ali MU Sjwed T Matenchuk BA Meyer S Impact of Postpartum Physical Activity on Maternal Anthropometrics: A Systematic Review and Meta–Analysis. Br J Sports Med (2025) 59:605–17. 10.1136/bjsports-2024-108449 40118514

[46] Pongpanit K Dayan N Janaudis–Ferreira T Roig M Spahija J Bertagnolli M . Exercise Effects on Maternal Vascular Health and Blood Pressure During Pregnancy and Postpartum: A Systematic Review and Meta–Analysis. Eur J Prev Cardiol (2024) 31:1606–20. 10.1093/eurjpc/zwae165 38711399

[47] Watkins VY Zhao P Frolova AI Carter EB Kelly JC Odibo AO The Association Between First Trimester Physical Activity Levels and Perinatal Outcomes. Am J Obstet and Gynecol MFM (2024) 6:101534. 10.1016/j.ajogmf.2024.101534 39490897 PMC11798543

[48] Apata T Samuel D Valle L Crimmins SD . Type 1 Diabetes and Pregnancy: Challenges in Glycemic Control and Maternal–Fetal Outcomes. In: Seminars in Reproductive Medicine. Thieme Medical Publishers, Inc. (2024). 10.1055/s-0044-1791704 39379044

[49] Hailu M Amare Tesfa N Nigatu A Tunta A Seyoum Z Derbew T . Physical Activity During Pregnancy and Pregnancy Related Compli– Cation. Scientific Rep (2025) 15:8980. 10.1038/s41598-025-94492-2 40089652 PMC11910504

[50] Acosta–Manzano P Flor–Alemany M Van Poppel MN Coll–Risco I Segura–Jiménez V Stanford KI Concurrent Exercise Training During Pregnancy Is Related to More Favourable Maternal Lipid Levels when IL–8 Increases. J Sports Sci (2025) 43:308–22. 10.1080/02640414.2025.2456384 39902852

[51] Filipec M Ðurin MJ . Thermoregulation and Endocrine Response During Exercise in Pregnancy. Physiologia (2025) 5:2. 10.3390/physiologia5010002

[52] Heljezovic´ Š Lucˇovnik M Verdenik I Šc´epanovic´ D . Association Between Regular Physical Activity During Pregnancy and Perinatal Outcomes: A Population–Based Cohort Study. Eur J Obstet and Gynecol Reprod Biol (2025) 26:100380. 10.1016/j.eurox.2025.100380 PMC11986463 40226794

[53] Nay K Smiles WJ Kaiser J McAloon LM Loh K Galic S Molecular Mechanisms Underlying the Beneficial Effects of Exercise on Brain Function and Neurological Disorders. Int J Mol Sci (2021) 22:4052. 10.3390/ijms22084052 33919972 PMC8070923

[54] Barker DJ . The Fetal and Infant Origins of Adult Disease. BMJ: Br Med J (1990) 301:1111. 10.1136/bmj.301.6761.1111 2252919 PMC1664286

[55] Barker DJ . The Developmental Origins of Adult Disease. J Am Coll Nutr (2004) 23:588S–595S. 10.1080/07315724.2004.10719428 15640511

[56] Hicks LE Graf MD Yeo S . Prenatal Exercise and Its Effects on Postpartum Mental Health: Systematic Review and Meta–Analysis. J Archiv Women’s Mental Health (2024). p. 1–10. 10.1007/s00737-024-01525-2 PMC12122183 39508925

[57] Evenson KR Mottola MF Artal R . Review of Recent Physical Activity Guidelines During Pregnancy to Facilitate Advice by Health Care Providers. Obstetrical and Gynecol Surv (2019) 74:481–9. 10.1097/OGX.0000000000000693 31418450

[58] Okafor UB Goon DT . Physical Activity Advice and Counselling by Healthcare Providers: A Scoping Review. Healthcare (2021) 9(5):609. 10.3390/healthcare9050609 34069474 PMC8159082

[59] Dolatabadi Z Amiri–Farahani L Ahmadi K Pezaro S . Barriers to Physical Activity in Pregnant Women Living in Iran and Its Predictors: A Cross Sectional Study. BMC Pregnancy and Childbirth (2022) 22:815. 10.1186/s12884-022-05124-w 36333661 PMC9636628

[60] Movitz S Mayer R Dingwall A . Incorporating Equity into Maternal Telehealth. J Georgetown Med Rev (2022) 6. 10.52504/001c.37490

[61] Paulden M Palmer S Hewitt C Gilbody S . Screening for Postnatal Depression in Primary Care: Cost Effectiveness Analysis. Bmj (2009) 339. 10.1136/bmj.b5203 20028779 PMC2797050

[62] Camacho EM Shields GE . Cost–Effectiveness of Interventions for Perinatal Anxiety and/or Depression: A Systematic Review. BMJ open (2018) 8:e022022. 10.1136/bmjopen-2018-022022 30099399 PMC6089324

[63] Slade SC Dionne CE Underwood M Buchbinder R . Consensus on Exercise Reporting Template (CERT): Explanation and Elaboration Statement. Br J Sports Med (2016) 50:1428–37. 10.1136/bjsports-2016-096651 27707738

[64] Dalbo VJ Carron MA . A Comparison of Physical Activity and Exercise Recommendations for Public Health: Inconsistent Activity Messages Are Being Conveyed to the General Public. Sports (2024) 12:335. 10.3390/sports12120335 39728875 PMC11840285

[65] Duchette C Perera M Arnett S White E Belcher E Tinius R . Benefits of Resistance Training During Pregnancy for Maternal and Fetal Health: A Brief Overview. Int *J Women’s Health* (2024). p. 1137–47. 10.2147/IJWH.S462591 PMC11193983 38912201

[66] Côté EJ Benton M Gardner R Tribe R . Balancing Benefits and Risks of Exercise in Pregnancy: A Qualitative Analysis of Social Media Discussion. BMJ Open Sport and Exerc Med (2024) 10:e002176. 10.1136/bmjsem-2024-002176 39415883 PMC11481124

[67] Haakstad LA Vistad I Sagedal LR Lohne–Seiler H Torstveit MK . How Does a Lifestyle Intervention During Pregnancy Influence Perceived Barriers to Leisure–Time Physical Activity? The Norwegian Fit for Delivery Study, a Randomized Controlled Trial. BMC pregnancy and childbirth (2018) 18:127. 10.1186/s12884-018-1771-8 29724165 PMC5934849

[68] Davenport MH Ruchat SM Garcia AJ Ali MU Forte M Beamish N Canadian Guideline for Physical Activity, Sedentary Behaviour and Sleep Throughout the First Year Post Partum. Br J Sports Med (2025) 59:515–26. 10.1136/bjsports-2025-109785 40139673

[69] May LE Moss SJ Szumilewicz A Santos–Rocha R Shojaeian NA . Barriers and Facilitators of Physical Activity in Pregnancy and Postpartum Among Iranian Women: A Scoping Review. Healthcare (2024) 12(23):2416. 10.3390/healthcare12232416 39685041 PMC11640986

[70] Santos–Rocha R . Exercise and Physical Activity During Pregnancy and Postpartum. Evidence–Based Guidel (2022) 2.

[71] Bull FC Al–Ansari SS Biddle S Borodulin K Buman MP Cardon G World Health Organi– Zation 2020 Guidelines on Physical Activity and Sedentary Behaviour. Br J Sports Med (2020) 54:1451–62. 10.1136/bjsports-2020-102955 33239350 PMC7719906

[72] Haskell WL Lee IM Pate RR Powell KE Blair SN Franklin BA Physical Activity and Public Health: Updated Recommendation for Adults from the American College of Sports Medicine and the American Heart Association. Circulation (2007) 116:1081–93. 10.1161/CIRCULATIONAHA.107.185649 17671237

[73] Yang Y Wang T Wang D Liu M Lun S Ma S Gaps Between Current Practice in Perinatal Depression Screening and Guideline Recommendations: A Systematic Review. Gen Hosp Psychiatry (2024) 89:41–8. 10.1016/j.genhosppsych.2024.04.011 38733723

[74] Kendall–Tackett KA . Screening for Perinatal Depression: Barriers, Guidelines, and Measurement Scales. J Clin Med (2024) 13:6511. 10.3390/jcm13216511 39518650 PMC11546415

[75] Liu W Li W Wang Y Yin C Xiao C Hu J Comparison of the EPDS and PHQ–9 in the Assessment of Depression Among Pregnant Women: Similarities and Differences. J Affective Disord (2024) 351:774–81. 10.1016/j.jad.2024.01.219 38290581

[76] Pascal R Casas I Genero M Nakaki A Youssef L Larroya M Maternal Stress, Anxiety, Well–Being, and Sleep Quality in Pregnant Women Throughout Gestation. J Clin Med (2023) 12:7333. 10.3390/jcm12237333 38068385 PMC10707410

[77] McCarthy M Houghton C Matvienko–Sikar K . Women’s Experiences and Perceptions of Anxiety and Stress During the Perinatal Period: A Systematic Review and Qualitative Evidence Synthesis. BMC Pregnancy and Childbirth (2021) 21:811. 10.1186/s12884-021-04271-w 34865642 PMC8647378

[78] Pan X Chen Y Chen C Chen J Wang J Chen Y Dual Trajectory of Insomnia and Depressive Symptoms in Women from Early Pregnancy to 6 Months Postpartum: A Prospective Cohort Study. BMC Pregnancy and Childbirth (2025) 25:582. 10.1186/s12884-025-07649-2 40382558 PMC12085819

[79] Stefana A Cena L Trainini A Palumbo G Gigantesco A Mirabella F Screening for Antenatal Maternal Depression: Comparative Performance of the Edinburgh Postnatal Depression Scale and Patient Health Questionnaire. Annali dell’Istituto Superiore di Sanità (2024) 60:55–63. 10.4415/ANN_24_01_08 38920259

