# Supplementary material for: Impact of Exercise Dose–Response on Maternal Mental Health and Perinatal Depression Prevention: A Systematic Review and Meta–Analysis
Source: Int J Public Health. 2025 Nov 21;70:1608940. doi: 10.3389/ijph.2025.1608940 (PMC12679042; doi:10.3389/ijph.2025.1608940)
Supplement: Supplementary file 1 [file Supplementaryfile1.docx]

Supplementary file 1: Therapeutic quality of exercise programs assessed using the i–CONTENT tool. (Chile. 2024-2025).

| **Study** | **Patient selection** | **Dosage** | **Type** | **Qualified supervision** | **Outcome assessment** | **Program safety** | **Adherence** | **Overall rating** **^b^** |
| --- | --- | --- | --- | --- | --- | --- | --- | --- |
| Coll et al. [[32]](#_bookmark37) | + | + | + | + | + | + | – | High |
| Daley et al. [[33]](#_bookmark38) | + | + | + | ND | + | + | – | Moderate |
| Davis et al. [[34]](#_bookmark39) | + | + | + | + | + | + | + | High |
| Duchette et al. [[35]](#_bookmark40) | + | + | + | + | + | + | ND | High |
| Kim et al. [[36]](#_bookmark41) | + | + | + | ND | + | ND | ND | Moderate |
| Mohammadi et al. [[37]](#_bookmark42) | + | + | + | ND^a^ | + | ND | – | Moderate |
| Özkan et al. [[38]](#_bookmark43) | + | + | + | ND | + | ND | ND | Moderate |
| Rong et al. [[39]](#_bookmark44) | + | + | + | + | + | + | + | High |
| Yang et al. [[40]](#_bookmark45) | + | + | + | ND^a^ | + | + | ND | Moderate |

**Legend:** (+): Criteria met / low risk of ineffectiveness. (–): Criterion not met / high risk of ineffectiveness. ND: No details provided.

a Intervention delivered by non–specialist or insufficient qualification details.

b The overall rating is based on the number of items rated as ’–’ or ’ND’. Studies with 0–1 such elements were considered of high quality; 2 to 3 elements, moderate quality; 4 or more, low quality. This classification reflects the judgment of the authors and should be interpreted with caution. The readers are encouraged to critically assess the strengths and weaknesses of each study individually.

Supplementary file 2: Funnel plot showing potential publication bias. (Chile. 2024-2025).


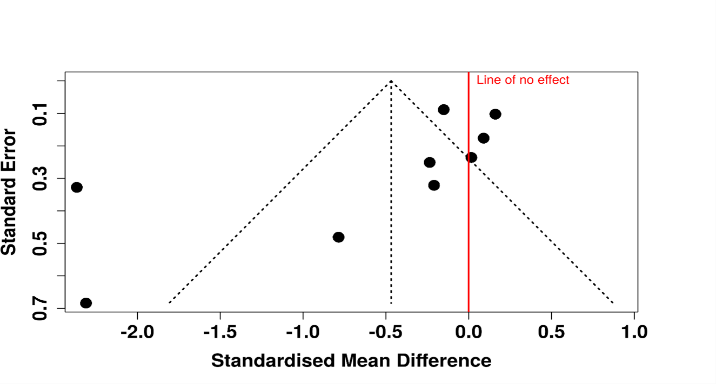


Supplementary file 3: Summary of findings table—GRADE assessment – Part 1. (Chile. 2024-2025).

| **Study** | *№* **of studies** | **Study design** | **Risk of bias** | **Inconsistency** | **Indirectness** | **Imprecision** | **Other considerations** |
| --- | --- | --- | --- | --- | --- | --- | --- |
| Coll et al. [[32]](#_bookmark37) | 9 | randomized trials | very serious | not serious | not serious | serious | none |
| Daley et al. [[33]](#_bookmark38) | 9 | randomized trials | not serious | not serious | not serious | serious | strong association |
| Davis et al. [[34]](#_bookmark39) | 9 | randomized trials | not serious | not serious | not serious | serious | strong association |
| Duchette et al. [[35]](#_bookmark40) | 9 | randomized trials | not serious | not serious | not serious | serious | strong association |
| Kim et al. [[36]](#_bookmark41) | 9 | randomized trials | not serious | not serious | not serious | serious | strong association |
| Mohammadi et al. [[37]](#_bookmark42) | 9 | randomized trials | not serious | not serious | not serious | serious | none |
| Özkan et al. [[38]](#_bookmark43) | 9 | randomized trials | not serious | not serious | not serious | not serious | very strong association |
| Rong et al. [[39]](#_bookmark44) | 9 | randomized trials | not serious | not serious | not serious | not serious | none |
| Yang et al. [[40]](#_bookmark45) | 9 | randomized trials | serious | not serious | not serious | not serious | none |

Supplementary file 4: Summary of findings table—GRADE assessment – Part 2. (Chile. 2024-2025).

| **Study** | **Exercise** | **Control** | **Relative (95% CI)** | **Absolute (95% CI)** | **Certainty** | **Importance** |
| --- | --- | --- | --- | --- | --- | --- |
| Coll et al. [[32]](#_bookmark37) | 192 | 387 | – | MD 0.6 lower (1.3 lower to 0.1 higher) | Very low | CRITICAL |
| Daley et al. [[33]](#_bookmark38) | 189 | 194 | – | MD 0.37 higher (0.59 lower to 1.33 higher) | High | CRITICAL |
| Davis et al. [[34]](#_bookmark39) | 20 | 19 | – | MD 0.09 lower (0.38 lower to 0.2 higher) | High | CRITICAL |
| Duchette et al. [[35]](#_bookmark40) | 10 | 9 | – | MD 2.23 lower (4.1 lower to 0.36 lower) | High | CRITICAL |
| Kim et al. [[36]](#_bookmark41) | 8 | 8 | – | MD 3.1 lower (5.35 lower to 0.85 lower) | High | CRITICAL |
| Mohammadi et al. [[37]](#_bookmark42) | 36 | 36 | – | MD 0.08 higher (1.23 lower to 1.39 higher) | Moderate | CRITICAL |
| Özkan et al. [[38]](#_bookmark43) | 34 | 31 | – | MD 5.25 lower (6.37 lower to 4.13 lower) | High | CRITICAL |
| Rong et al. [[39]](#_bookmark44) | 32 | 32 | – | MD 0.87 lower (2.69 lower to 0.95 higher) | High | CRITICAL |
| Yang et al. [[40]](#_bookmark45) | 64 | 65 | – | MD 1.75 lower (3.2 lower to 0.3 lower) | Moderate | CRITICAL |
