# Supplementary material for: Impact of Exercise Dose–Response on Maternal Mental Health and Perinatal Depression Prevention: A Systematic Review and Meta–Analysis
Source: Int J Public Health. 2025 Nov 21;70:1608940. doi: 10.3389/ijph.2025.1608940 (PMC12679042; doi:10.3389/ijph.2025.1608940)
Supplement: Supplementary file 2 [file Supplementaryfile3.docx]

Supplementary file 3: Summary of findings table—GRADE assessment – Part 1. (Chile. 2024-2025).

| **Study** | *№* **of studies** | **Study design** | **Risk of bias** | **Inconsistency** | **Indirectness** | **Imprecision** | **Other considerations** |
| --- | --- | --- | --- | --- | --- | --- | --- |
| Coll et al. [[32]](#_bookmark37) | 9 | randomized trials | very serious | not serious | not serious | serious | none |
| Daley et al. [[33]](#_bookmark38) | 9 | randomized trials | not serious | not serious | not serious | serious | strong association |
| Davis et al. [[34]](#_bookmark39) | 9 | randomized trials | not serious | not serious | not serious | serious | strong association |
| Duchette et al. [[35]](#_bookmark40) | 9 | randomized trials | not serious | not serious | not serious | serious | strong association |
| Kim et al. [[36]](#_bookmark41) | 9 | randomized trials | not serious | not serious | not serious | serious | strong association |
| Mohammadi et al. [[37]](#_bookmark42) | 9 | randomized trials | not serious | not serious | not serious | serious | none |
| Özkan et al. [[38]](#_bookmark43) | 9 | randomized trials | not serious | not serious | not serious | not serious | very strong association |
| Rong et al. [[39]](#_bookmark44) | 9 | randomized trials | not serious | not serious | not serious | not serious | none |
| Yang et al. [[40]](#_bookmark45) | 9 | randomized trials | serious | not serious | not serious | not serious | none |
