# Supplementary figures and images for: Impact of Exercise Dose–Response on Maternal Mental Health and Perinatal Depression Prevention: A Systematic Review and Meta–Analysis
Source: Int J Public Health. 2025 Nov 21;70:1608940. doi: 10.3389/ijph.2025.1608940 (PMC12679042; doi:10.3389/ijph.2025.1608940)

Supplementary file 2: Funnel plot showing potential publication bias. (Chile. 2024-2025).


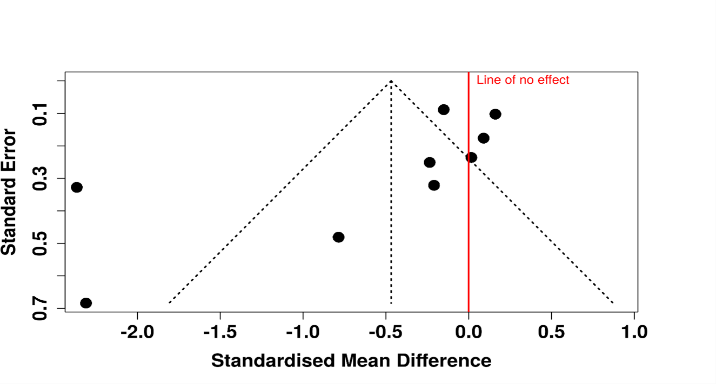

Supplement: Supplementary file 3 [file Supplementaryfile2.docx]
