# Supplementary material for: Impact of Exercise Dose–Response on Maternal Mental Health and Perinatal Depression Prevention: A Systematic Review and Meta–Analysis
Source: Int J Public Health. 2025 Nov 21;70:1608940. doi: 10.3389/ijph.2025.1608940 (PMC12679042; doi:10.3389/ijph.2025.1608940)
Supplement: Supplementary file 4 [file Supplementaryfile4.docx]

Supplementary file 4: Summary of findings table—GRADE assessment – Part 2. (Chile. 2024-2025).

| **Study** | **Exercise** | **Control** | **Relative (95% CI)** | **Absolute (95% CI)** | **Certainty** | **Importance** |
| --- | --- | --- | --- | --- | --- | --- |
| Coll et al. [[32]](#_bookmark37) | 192 | 387 | – | MD 0.6 lower (1.3 lower to 0.1 higher) | Very low | CRITICAL |
| Daley et al. [[33]](#_bookmark38) | 189 | 194 | – | MD 0.37 higher (0.59 lower to 1.33 higher) | High | CRITICAL |
| Davis et al. [[34]](#_bookmark39) | 20 | 19 | – | MD 0.09 lower (0.38 lower to 0.2 higher) | High | CRITICAL |
| Duchette et al. [[35]](#_bookmark40) | 10 | 9 | – | MD 2.23 lower (4.1 lower to 0.36 lower) | High | CRITICAL |
| Kim et al. [[36]](#_bookmark41) | 8 | 8 | – | MD 3.1 lower (5.35 lower to 0.85 lower) | High | CRITICAL |
| Mohammadi et al. [[37]](#_bookmark42) | 36 | 36 | – | MD 0.08 higher (1.23 lower to 1.39 higher) | Moderate | CRITICAL |
| Özkan et al. [[38]](#_bookmark43) | 34 | 31 | – | MD 5.25 lower (6.37 lower to 4.13 lower) | High | CRITICAL |
| Rong et al. [[39]](#_bookmark44) | 32 | 32 | – | MD 0.87 lower (2.69 lower to 0.95 higher) | High | CRITICAL |
| Yang et al. [[40]](#_bookmark45) | 64 | 65 | – | MD 1.75 lower (3.2 lower to 0.3 lower) | Moderate | CRITICAL |
